# Current endoscopic methods of radical therapy in early esophageal cancer

**Published:** 2015

**Authors:** A Mocanu, R Bârla, P Hoara, S Constantinoiu

**Affiliations:** * Surgery Clinic, “Sf. Maria” Clinical Hospital, Bucharest, Romania

**Keywords:** esophageal early cancer, radical therapy, endoscopy

## Abstract

During the last three decades, there has been an increasing incidence of the esophageal cancer at the global level, approx. 400,000 new esophageal cancers being currently diagnosed annually. This is the eighth leading cause of cancer incidence and the sixth leading cause of cancer death overall.

If we refer to the countries of Western Europe and North America, we could see an increase in the esophageal adenocarcinoma in detriment of squamous cancer. As for the Asian region, referring in particular to China and Japan, 9 out of 10 esophageal cancers are squamous cell carcinomas.

Considering that the incidence of gastric cancer in Japan is very high, the endoscopic screenings performed inevitably led to an increased rate of early detection of esophageal cancer, reaching approximately 20% of all esophageal cancers detected. This has led to the possibility of developing therapeutic endoscopic techniques with radical visa that we will describe while presenting comparative data from literature.

Currently, however, there are not enough data on the effectiveness of these types of therapies, compared to surgery, in order to be transformed into standard therapeutic endoscopic treatment for early esophageal cancer.

However, the combined therapy, resection/ endoscopic ablation + chemoradiotherapy, appears as an alternative to be taken into account.

**Abbreviations** EEC = esophageal early cancer, BE = Barrett’s esophagus, HGD = High-grade dysphagia, EUS = Ultra sound endoscopy, CT = Computer tomograph, UGE = Upper gastro endoscopy, PET-CT = Positron Emission Tomography, FNAB = Fine needle aspiration biopsy, EMR = Esophageal mucosal resection, ESD = Esophageal submucosal dissection, SCC = Squamous cellular cancer, PCT = Poli-chemotherapy, RT- Radio-therapy.

## Introduction

Esophageal cancer is one of the most aggressive digestive tumors, the average survival in about 5 years being of 10% even in specialized centers. In contrast, the 5 years survival in some early treated cancers can reach 92-93%, in 25 years reaching only 48%, more than half of the deaths being due to neoplastic recurrence.

Although HGD and EC can be treated by surgery while using radical esophagectomy, high postoperatory mortality and morbidity led to a raising interest in complementary or alternative therapies to be used. New endoscopic techniques for esophageal neoplastic lesions began to be applied after almost two decades. At this point, from the existing data, it is not clear which of the ablative or resection techniques can be considered optimal. Moreover, the risk of tumor recurrence or incomplete resections of tumor tissue remain the main problems of endoscopic therapies, which may find an answer in the present comparative trials.

In principle, considering the EC staging, endoscopic therapies can be classified into methods with curative visa and palliation methods. In terms of the methods used, they are classified in endoscopic resection techniques and ablation techniques.

Concerning the population global aging and thus, the increase in the number of patients who will not bear surgery, PCT and/ or radiation, or they do not want this, especially because of deficiencies and associated pathology; the endoscopic ablative techniques with palliation purposes represent the alternative.

Curative endoscopic techniques may be used only in EC stages T1a and T1b.

Stage T1a includes the following substages:

- T1aM1 represented by epithelial lesions,

- T1a M2 including the invasion of lamina propria and,

- T1aM3 invasion of the muscularis mucosae reaching 2000nm depth.

Stage T1b includes:

- T1bSm1 superficial submucosal invasion,

- T1bSm2 central submucosal invasion and

- T1bSm3 representing deep submucosal invasion in esophageal wall [**1**,**2**].

Depending on the early stage of neoplasia and, implicitly, the possibility of N metastasis, the type of endoscopic therapy can be chosen and the long-term prognosis can be established. The stage T1aM has a minimal risk of lymphatic invasion, while T1b shows a significant probability of lymph node invasion up to 21% in Sm1 and 56% in Sm3 [**3**]. Disease recurrence is cited between 1.2% at 5 years for T1a and 19.5% in T1b also at 5 years [**4**]. The perivascular invasion and low cell grading (G3) in early stages also represent an increased risk for metastasis. Thus, the need for a careful selection of patients who will undergo endoscopic procedures with radical visa is obvious.

**Patient selection**

Considering the above, the upper gastrointestinal endoscopy, accompanied by serial biopsies +/- magnification and chromoendoscopy, is a standard investigation in EC early detection and staging [**4**]. The size of detected lesions should be of about 20mm in surface and between T1M1 and T1M3 in depth. It is desirable that the circumferential extension occupies maximum 2/ 3 of the esophageal lumen, complete circumferential lesions endoscopically treated leading to stenosis at a rate of 37% which can benefit from dilations, yet more difficult than the peptic ones [**5**]. 

Staging modalities for patients selection included as standard CT and EUS, investigation that can give the most accurate assessments in terms of T and N staging locally with 85% accuracy for T and 81% for N [**6**,**7**], more difficult in the case of superficial cancers [**8**].

The early studies performed in 1987 showed a precision in EUS diagnosis of stage T1a and T1b of only 29%, and 42% for T2 [**9**].

At the moment, a study on 42 patients diagnosed by EUS and undergoing surgery, showed a 100% diagnosis for T and 89% for N [**10**]. 

The association of other imaging tests like PET-CT (prohibited as standard method because of high price) and transesophageal FNAB with EUS, could increase the diagnosis accuracy and T and N staging compared to EUS alone [**11**,**12**].

Another discussed technique of avoiding substaging N for T1b and calculating the probability of lymph node metastasis, is the pathological assessment of depth of invasion in esophageal wall on specimens obtained by EMR [**13**]. The pathological result accuracy is superior on specimens obtained by EMR than serial biopsies of the EDS [**14**].

In addition, in the sense of detecting the probability of micro metastasis in the lymph nodes in T1 stages and establishing the appropriate therapeutic management, the molecular or biological assessment methods of predictively determining IHC expression of desmogleine1 or mRNA expression of telomerase by reverse transcriptase-polymerase chain reaction (RT-PCR) are not negligible. At present, these tests have not been usually performed to establish the prognosis in esophageal neoplasia.

**Therapeutic endoscopic methods with curative visa**

In early EC, the endoscopic techniques can be divided into ablative techniques and resection techniques. The resection methods consist in EMR and ESD. These two types of endoscopic therapies, versus the ablative ones offer the advantage of obtaining a mucosal specimen that may result, by pathological analysis, in a better and more accurate staging [**15**].

**EMR**

**Fig. 1:**
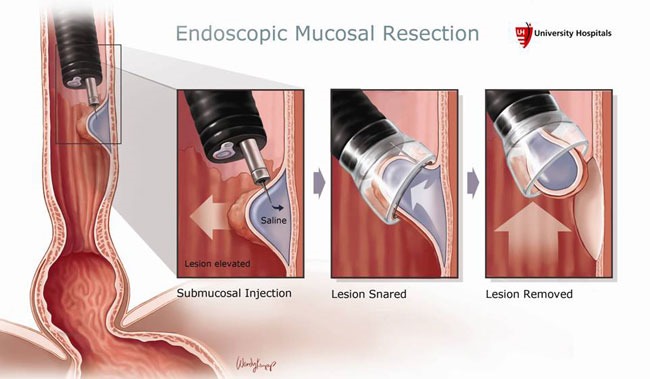
Endoscopic Mucosal Resection

 The idea of using EMR for the excision of superficial neoplasia originated in Japan, where the incidence of esophageal EC detection is high due to the screening programs for the early diagnosis of gastric cancer. Unlike the ablative techniques that destroy the neoplastic tissue, EMR performs its excision, enabling the pathologist to make a better staging. At present, there are many technical options, but the method principle comprises the injecting of dilute epinephrine or saline at the submucosal level under the suspicious lesion, vacuuming the targeted area by using the variceal ligation model and its electro-resection using a loop [**16**] (**Fig. 1**).

A study on 70 patients with HGD and EC whereas EMR was performed, caused a local remission of 90%, but long-term lesions (3 years) were detected in 30% of the patients [**17**]. Other authors report a healing of 91% to 98% in T1a stage neoplasms [**18**,**19**].

The possible complications of EMR consist in bleeding - 10% [**20**-**22**], esophageal perforation, 3% [**23**-**25**] and stenosis. Stenosis severity varies given the area and depth of resection, which can occur in 37% of the cases [**26**].

EMR advantages mainly consist in the possibility of providing precious esophageal tissue samples to correct the T and N staging of EC [**20**] and, in HGD and EC, in T1a stages, has a curative value.

Comparing ESD to EMR is suitable in the same situations, but is superior to EMR in the resection of larger dysplastic lesions of more than 2cm also in T1b stages. Japanese statistics on ESD in the treatment of SCC, shows a 100% “en bloc” resections, with a cure rate of 80% [**27**,**82**]. Compared with EMR, the accuracy of the method is rated at the value of 100% for the “en bloc” resection, for ESD by using a Hook or Flex-type electrolancet vs. only 53% for EMR and a relapse rate of only 0.9%, compared to 9.8% with ESD, given EMR [**28**].

Complications of ESD are represented by perforations between 2-5% and a frequency of stenoses between 5-17% [**28**,**29**]. Unfortunately, the data containing information relative to ESD are relatively poor, mainly coming from the Asian region, especially Japan. The explanation lies in the relatively low frequency of SCC in North America and Western Europe versus esophageal adenocarcinoma, in contradiction with the high percentage of squamous cell cancer observed in Japan, ESD being thus sporadically practiced in the US.

**Ablation techniques**

Ablative techniques used in the treatment of BE and esophageal SCC include:

- photodynamic therapy (PDT)

- argon coagulation (APC)

- radiofrequency ablation (RFA)

- cryotherapy 

- thermocoagulation.

All these types of endoscopic therapy can be used alone or in combination with other types of endoscopic therapeutic methods, usually a resection-type one, as first or secondary method.

**PDT**

Represents a two-stage ablation technique which does not use temperature, yet it relies on oral or i.v. administering a photosensitive substance with tropism for the neoplastic tissue or BE-type tissue [**30**]. Further stimulation of the substance with a beam of light of a precise frequency (630 nm) leads, after a photochemical reaction, to the release of oxygen radicals that will cause the target-cell destruction.

**Fig. 2:**
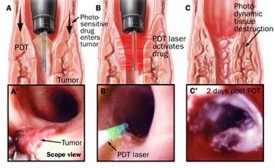
Tissue necrosis process of 8 to 10 days

The substance used is a sodium salt of porphyrin, the commercial preparation used in US as Photofrin. In Europe, it is used as a photosensitizing substance 5-aminolevulinic acid (5-ALA). Currently, there are no studies comparing the efficacy of two substances, but both remain at a fairly high price. Apparently, using 5-ALA would predispose slightly less at strictures as late complications of the method. Once the photosensitive substance stimulated, the tissue necrosis process takes about 8 to 10 days (**Fig. 2**).

Existing studies recommend the use of PDT as a single therapy in EC only after the assessment of pathological lesion depth, based on mucosal samples collected by EMR. Success rate reported in the use of PDT together with other secondary techniques in the treatment of T1 SCC tumors is of approx. 87% on a study conducted on a total of 38 patients, however with a relapse rate of 18% [**31**,**32**]. From the perspective of PDT use in Barrett's esophagus and early esophageal cancer, one of the earliest and largest studies was published by Overholt et al. [**31**, **33**]. These authors used a cylindrical inflatable balloon to deliver light in 101 patients treated with Photofrin. At a mean follow up of 4 years, 54% of the patients demonstrated a complete resolution of Barrett's metaplasia, whereas 78% resolved HGD and 48% of early cancers were deemed cured. Treatment-related stenosis occurred in 30% of the patients.

 Early complications specific to this method consist of ocular and cutaneous photosensitivity lasting for about 30 days due to the secondary concentration of the photosensitizing substance in the skin and retina. Other common complications are somewhat common to endoscopic ablative techniques and consist of chest pain, sore throat, nausea and rarely arrhythmias [**34**,**35**]. Late complications are the strictures and stenosis described in a proportion of 30-40% in a study on 131 patients [**36**]. Their treatment consisted of a successive dilatation (4 sessions on average). Monitoring the patients who have undergone this treatment is required by EDS in order to detect possible early relapse or recurrence in case of BE. It is also mandatory to advise potential patients who benefit from the endoscopic method on the possibility of tumor recurrence and on the alternative of surgical therapy checked in time, but encumbered by postoperative mortality and morbidity.

**Radiofrequency ablation (RFA)**

**Fig. 3:**
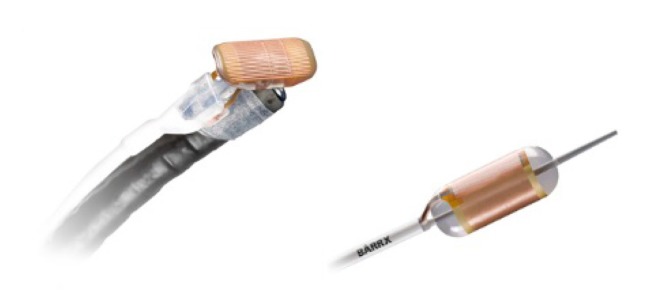
Applications lasting for about one second are carried out on the suspect area and around it

This relatively new method is used in the treatment of BE and HGD. From the technical point of view, a series of electrodes attached either to an endoscope or, more recently, to multi-shape balloons especially designed are used. The electrodes are connected to a source that emits high frequency currents (350-500 kHz) and, placed in direct contact with the esophageal mucosa, cause the destruction of the targeted area. The applications lasting for about one second are carried out on the suspect area and around it on a surface of approx. 1 cm, to decrease the remaining HGD areas as much as possible (**Fig. 3**).

The advantage of the method consists in the uniformity of the ablation in both, surface and depth. Therefore, on a study of 73 patients observed for one year and from whom a total number of approx. 4306 biopsies have been collected, deep remaining methaplasic epithelium (“buried” glands) was not observed as in PDT [**37**].

To check the depth of the ablation by using this procedure, a study was performed on a group of patients with HGD firstly treated with RFA and then operated. The HP examination of specimens of surgical excision showed that RFA ablation was performed uniformly up to muscularis mucosae level, with a success rate of approx. 90% [**38**]. At the same time, it seems that due to the uniformity of ablation, the frequency of stenotic complications of type is not as high as in the case of PDT.

**Argon ablation (APC)**

**Fig. 4:**
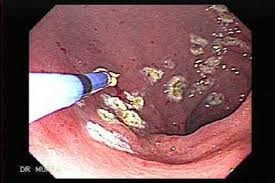
The “spray” coagulation used in the treatment of BE without dysplasia

This technique, which was commonly used in the treatment of liver surgery and in rectal tumors and ulcers post-radiotherapy, is based on tissue destruction by thermo-coagulation achieved by targeting a highly ionized argon jet with a mono-polar current on a probe that must not be exposed to the mucosal membrane. The “spray” coagulation is used in the treatment of BE without dysplasia, but the recurrence rate is high [**39**] (**Fig. 4**).

A study on a small number of patients-10, of whom 7 with HGD and 3 with EC treated by PCA and re-evaluated one year later showed that one patient still had HGD and another had EC. Another small series (3 patients) studied with EC who underwent APC showed a patient with lasting EC at 2 years.

**Laser therapy**

Using laser is another way of destructing the malignant tissue by using thermo-coagulation. The most common type of laser currently eligible for use is Nd: YAG having a wavelength of about 1064 nm, at an energy between 80 and 100 watt, some authors considering that applications of 40-50watt at a time length of 0.5 -1 seconds in 1-2 sessions would be enough [**40**]. Studies on the effectiveness of laser therapy are relatively few in HGD and EC, this type of therapy being used mainly as palliative endoscopic method in dysphagia in advanced esophageal cancer. However, a study on 14 patients with HGD and EC treated with Nd: YAG showed that, at 1 year, 22% of the subjects showed residual BE without HGD or cancer [**41**].

The advantages of APC, at least technically speaking, as compared to LASER therapy, are that the equipment is not so bulky, does not require special safety measures, and the tissue destruction temperatures required are lower than the ones while using LASER. This may have repercussions regarding the number and severity of complications such as pain, perforation or post-therapy stenosis. Another advantage is the depth of only 1-3mm at which the ablation therapy is produced, opposed to the laser therapy where it reaches up to 3-6mm. In a study on 42 patients treated with APC and then submitted to surgery, thermo-coagulation areas to the level of muscularis propria were found only in one case [**42**].

**Multipolar electrocoagulation**

The method also relies on producing thermal injury to the mucosa performed by using a transendoscopic probe. This type of ablation was used especially in BE without dysplasia. Existing studies showed the healing at a rate of approx. 75%, but a follow-up between 4 months and 3 years proved the existence of BE lesions between 0% and 27% [**43**,**44**]. These results demonstrated that repeated electrocoagulation sessions would be required to complete the healing of BE.

At the same time, it should be noted that this method is not recommended in the treatment of EC or HGD due to the small size of the probe that makes a correct ablation of the affected area practically impossible.

**Cryoablation**

Unlike most of the other ablation methods that use thermocoagulation for the destruction of pathological mucosal areas, it uses low temperatures obtained by applying a jet of liquid nitrogen introduced through a special transendoscopic catheter. A study on a group of 11 patients with LGD and HGD showed wound healing at 6 months [**45**]. Complications encountered are esophageal ulcers, mild chest pain and transient dysphagia, degree II-III Takita.

The preliminary results of the existing studies are encouraging, but are far from allowing the principle introduction of the method in the treatment of early esophageal cancer. Another problem of cryoablation, as well as of APT is the inconsistent application of the therapeutic agent, the accuracy of treatment practically depending on the skills and experience of the endoscopist. We can thus conclude that, due to the insufficient data, both LASER therapy and APC are not indicated as radical therapy in HDG and EC.

**Combined therapies**

We have shown above that in patients with long-BE, the correct resection cannot be performed by EMR. Combining EMR with ablative therapies has not been systematically studied, however, a study on 28 patients with macroscopic lesions of HDG or EC whereat EMR was performed, followed by PDT or APC, a remission was achieved in 26 of 28 patients at 4,5 months of treatment [**46**]. Yet in a follow-up of 1,5 years, from the patients with EMR and PDT, 19% had recurrent HGD and deep BE.

Comparing the results of another retrospective study, a group of 24 patients with EMR treated by EC + PDT, to a group of 64 patients treated by esophagectomy, it was proved that 83% of the first batch were “cancer free” at 1 year, compared to 100% of the second group. Yet what should be mentioned is the high rate of morbidity post-surgery of the second batch, versus 0% of the endoscopic treated batch [**47**]. In another study on 742 patients with TisN0M0 and T1N0M0, who were endoscopically and surgically treated (most of all by EMR), Dass et al. [**48**] showed a comparable survival to 5 years, (56 months for endoscopic treatment and 59 months for surgical treatment).

Zeneth also showed a superposable survival EMR/ surgery at 3 years with a higher morbidity, but in favor of surgery (0% EMR versus 39% surgery) [**49**].

Another recent study [**50**] showed that both the endoscopic therapy in BE and BE with HGD and in EC was more efficient in terms of cost-effectiveness.

Another possibility of treatment is EMR followed by RT and PCT for the prophylaxis of possible secondary lymph-node metastasis, the protocol consisting in EMR + 5FU + Cis - 5 days at a 3 weeks interval. A comparative study prospectively evaluated the results obtained by EMR followed by PCT or RT in T1b stage, and patients with the same stage of disease who have been surgically treated [**51**]. The survival of patients for 5 years was equal in the two groups. Benefits of EMR + RT +/- PCT are:

- a too aggressive treatment can be avoided 

- for CSS submucosal (SM) the resection can be completed, possibly by ESD

- low doses of radiation can be reduced up to 40Gy [**52**].

## Conclusions

1. The main purpose of endoscopic therapy in EC and HGD is to lead to the healing with a minimal tissue sacrifice and by avoiding post-operative comorbidities.

2. This type of therapy is addressed both to patients with HGD and EC, and to patients with major surgical contraindications.

3. The selection and, implicitly, the pre operative staging of patients, especially for N, are mandatory.

4. For the T1b stage, the surgical treatment continues to be the standard. At present, there are not enough long-term studies allowing a comparison between surgery and therapeutic endoscopy +/- adjuvant therapy.

5. The early detection of esophageal neoplasia by screening programs represents the “keystone” in the success of endoscopic radical therapy.
